# A Rare Case of Oropharyngeal and Nasopharyngeal Stenosis Secondary to Self‐Inflicted Gunshot Wound

**DOI:** 10.1155/crot/6898723

**Published:** 2026-05-20

**Authors:** Eric Tran, Jordan Kankam, Daniel J. Spangler, Drew H. Smith, Joshua Demke

**Affiliations:** ^1^ School of Medicine, Texas Tech University Health Sciences Center, Lubbock, Texas, USA, ttuhsc.edu; ^2^ Department of Otolaryngology and Head & Neck Reconstructive Surgery, Texas Tech University Health Sciences Center, Lubbock, Texas, USA, ttuhsc.edu

**Keywords:** head and neck, nasopharynx, oropharynx, stenosis, trauma

## Abstract

Synechiae are abnormal adhesions or scar tissue that develop between two normally separate anatomical surfaces. Here, we present a rare case in which oropharyngeal and nasopharyngeal synechiae resulted in partial stenosis and functional impairments in speech, swallowing, and airway patency. The patient underwent surgical lysis of the scar bands, in which the bilateral bands connecting the tongue base to the posterior palate and pharyngeal wall were released using needle‐tip Bovie cautery. Follow‐up demonstrated significant improvement in breathing and swallowing. This case highlights the importance of understanding the healing process following traumatic injury to the oral and nasopharyngeal mucosa to better recognize and manage postoperative complications arising from abnormal adhesions. Further studies should investigate predisposing factors that increase susceptibility to such formations, as well as techniques and practices to prevent complications similar to the one presented here.

## 1. Introduction

Synechiae are abnormal adhesions or scar tissue that develop between two normally separate anatomical surfaces [1]. In rare instances, synechiae can result in stenosis between the nasal and throat passages (the nasopharynx and oropharynx, respectively), leading to airway constriction, impaired speech articulation, and difficulty swallowing and chewing, ultimately impacting quality of life [2]. Surgical intervention, such as excising the band of scar tissue, can help restore normal function and improve these deficits. Here, we present a unique case of a patient with synechiae extending from the nasopharynx to the soft palate and from the soft palate to the tongue, secondary to a self‐inflicted gunshot wound.

## 2. Case

A 72‐year‐old female presented to the ENT trauma service following a self‐inflicted gunshot wound to the face, resulting in trauma to the oral, oropharyngeal, and nasopharyngeal cavities. She underwent a cricothyroidotomy by EMS prior to arrival at the hospital for airway securement. Following initial trauma assessment, she proceeded to the operating room for direct laryngoscopy, which showed complex lacerations of her posterior pharyngeal wall and tongue base. CT imaging revealed associated soft tissue emphysema and only a minimally displaced fracture of the horizontal plate of the palatine bone, with no other fractures of the facial, cervical, or cranial bones or intracranial injury. Direct laryngoscopy had shown complex, piecemeal lacerations of the tongue, extensive laceration of the soft palate, and extensive laceration of the posterior pharyngeal wall. Closure of the more anterior tongue lacerations was accomplished with Vicryl sutures. However, given the extent and irregularity of the tissue defects and the anatomically challenging location, much of the posterior and distal aspects of the wound were not amenable to primary closure or immediate reconstruction and were left to heal by secondary intention. While this approach minimized tension and preserved tissue viability, it likely contributed to aberrant healing and subsequent adhesion formation, given the close apposition of mucosal surfaces within the confined upper aerodigestive tract. To secure a safe and patent airway, she underwent formalization of her cricothyroidotomy to a tracheostomy. For establishment of nutritive enteral access and to bypass the wounds to allow for healing, she also underwent gastrostomy tube (G‐tube) placement. Her postoperative inpatient course was uneventful; she was able to tolerate an oral pureed diet and supplemental feeds through her G‐tube during her stay. The patient’s tracheostomy was decannulated after 2 weeks of admission following a successful capping trial, and she was later discharged with outpatient ENT follow‐up. She also underwent G‐tube removal 1 month after discharge, as she was able to tolerate appropriate oral intake.

**FIGURE 1:**
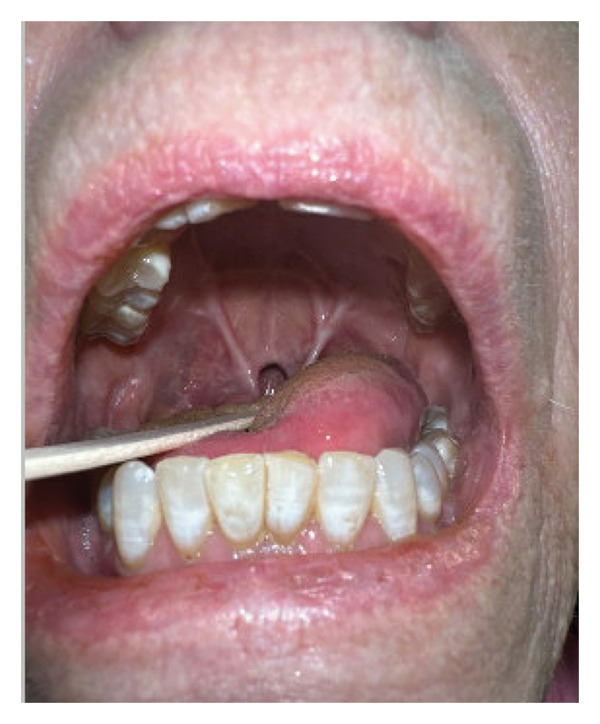
Coronal view of severe oropharyngeal synechiae extending from the nasopharynx to the soft palate and from the soft palate to the tongue, resulting in near‐total circumferential stenosis.

Five months later, the patient presented to the otolaryngology clinic with oropharyngeal and nasopharyngeal synechiae resulting in near‐total circumferential stenosis of her oropharynx at the level of the tongue base. The tracheostomy and G‐tube had been removed, but the patient stated that she was experiencing difficulties with breathing and swallowing caused by the synechiae that formed over time, as well as changes to her speech. On physical examination, the patient had synechiae that formed from the nasopharynx to the soft palate and from the soft palate to the tongue, with one large opening centrally and two small ports laterally, resembling the irregularity of the tissues that had been left to heal previously (Figure 1). Given these clinical findings, the risks, benefits, and alternatives to surgical intervention were discussed; the patient expressed her desire to undergo surgical lysis of adhesions. She was deemed a surgical candidate, and preparations were made for outpatient surgical planning.

Three months later, the patient underwent the operation. Initially, anesthesia noticed that the patient’s airway status was a Mallampati Classification IV.

With this, in the operating room after discussion with the anesthesia team, the patient underwent awake fiberoptic nasal intubation given the near‐complete oropharyngeal stenosis and limited mouth opening. This began with the administration of a combination Neosynephrine/lidocaine topical spray to anesthetize and decongest the upper aerodigestive tract. Next, a narrow‐caliber flexible fiberoptic endoscope was directed through the nose and then nasopharyngeal inlet to visualize the airway from the nasal inlet to the level of the subglottis, followed by the advancement of an endotracheal tube through the glottic inlet using the Seldinger technique. This was uneventful without significant complications, as evidenced by confirmatory end‐tidal CO2 capnography.

**FIGURE 2:**
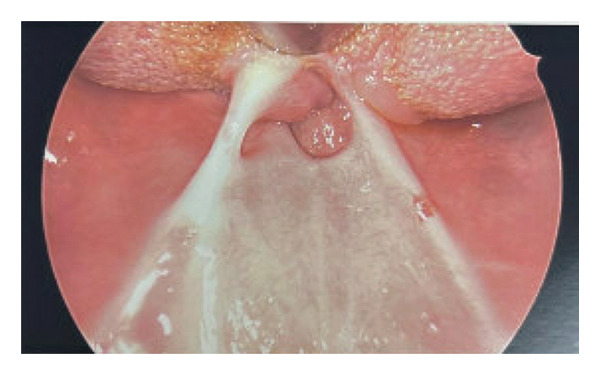
Preoperative endoscopic view illustrating bilateral scar bands connecting the tongue base to the posterior pharyngeal wall, forming a significantly restricted central airway inlet.

**FIGURE 3:**
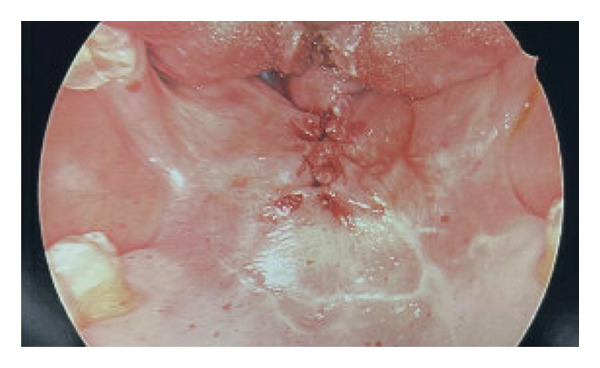
Postoperative view immediately following surgical lysis of the scar bands and layered repair of the soft palate dehiscence.

In addition, recognizing the high risk for a “cannot intubate, cannot oxygenate” (CICO) scenario, the team had surgical equipment immediately available in the operating room for an awake revision tracheostomy, prepared in the event of sudden laryngospasm or bleeding in the restricted surgical field. The reason for awake fiberoptic nasal intubation over other methods was due to the severity of the synechiae and limited mouth opening, which would have made direct visualization or blind techniques unsafe. Furthermore, the previous tracheostomy site was evaluated preoperatively to ensure distal airway patency, supporting safe passage for the fiberoptic scope. Her oropharyngeal synechiae appeared unchanged from the previous in‐clinic examination; much of her nasopharynx and oropharynx were stenotic due to circumferential synechiae. Nonetheless, intubation proceeded smoothly, and upon fiberoptic examination, the tissues of her hypopharynx and larynx distal to the synechiae appeared widely patent and free of scarring. After sterile prep and draping, bilateral scar bands connecting her tongue base to her posterior palate and pharyngeal wall (Figure 2) were released with needle‐tip Bovie cautery, reestablishing a much more widely patent upper aerodigestive tract passage. The release of the velar tissue resulted in a midline soft palate dehiscence that was then addressed by closure in layers with 4‐0 Vicryl sutures for the nasal layer, 4‐0 Vicryl sutures for the oral layers, and interrupted 4‐0 PDS sutures to create a normalized soft palate (Figure 3). Lastly, the oral cavity and oropharynx were suctioned, and the patient was reversed from anesthesia and awakened prior to extubation.

The patient followed up at the otolaryngology clinic 2 weeks postoperatively with significant improvement in breathing, swallowing, and speech. Flexible nasopharyngolaryngoscopy revealed a widely patent oropharyngeal and nasopharyngeal airway with well‐healed mucosa and no evidence of recurrent adhesions or restenosis. The soft palate repair was intact without dehiscence. The physical examination showed that the oropharyngeal wall healed with no dehiscence, with the tongue being free from any attachment to the palate or pharyngeal wall. Speech was intelligible without clinically significant hypernasality, and there was no evidence of velopharyngeal insufficiency on examination. The patient was also tolerating a regular diet without subjective issues with feeding. She followed up again 8 weeks postoperatively, confirming sustained improvement, with the oropharyngeal wall healed and the tongue free from any attachment to the palate or pharyngeal wall. Due to geographical constraints and ongoing psychiatric care, the patient has not been physically present for further follow‐up; however, at the 8‐week mark, no reformation of synechiae had occurred. To date, no reformation of synechiae has occurred.

## 3. Discussion

Oropharyngeal and nasopharyngeal synechiae are a highly rare complication of traumatic or iatrogenic injuries of the upper aerodigestive tract. Synechiae can form between mucosalized surfaces as they heal and anneal to one another during a period of tissue growth or regrowth. In this case, the patient developed synechiae from the nasopharynx to the soft palate and from the soft palate to the tongue during her recovery phase secondary to her self‐inflicted gunshot wound.

The most likely mechanism behind the synechiae in this patient appears to be due to prolonged apposition of raw mucosal surfaces during secondary intention healing, allowing opposing epithelium to adhere. Contributing risk factors that may have led to this problem could be due to possible tissue defects, limited mouth opening, immobility of the soft palate, or delayed tissue remodeling. This mechanism is consistent with literature where adhesions form through maturation of fibrin bridges to fibrous scar tissue, mediated by fibroblasts and myofibroblasts under the influence of TGF‐B [1–3]. Prolonged delay in having the synechiae scar bands lysed or released could potentially lead to progressive speech impairments that affect quality of life [4]. For example, velopharyngeal insufficiency, a disorder of the velopharyngeal sphincter or valve, might manifest as hypernasal speech, increased nasal resonance, and nasal emission during speech due to the soft palate’s inability to form an effective seal. Over time, these potential consequences can result in incompetence from neurologic dysfunction or mislearning due to acquired articulation errors [5]. Additionally, the small inlet formed by the scar tissues could act as an obstruction, potentially leading to sleep‐disordered breathing, obstructive sleep apnea, stridor, or wheezing [4]. Despite an advanced PubMed search using the terms “((((pharyngeal) OR (oropharyngeal)) OR (nasopharyngeal)) AND ((synechiae) OR (scar))) AND (trauma),” we were unable to identify prior reports of delayed traumatic oropharyngeal and nasopharyngeal synechiae following gunshot injury.

From a clinical perspective, this case highlights several preventative and management considerations involved with synechiae formation. Early endoscopic surveillance could provide initial detection of mucosal contact or early fibrin deposition, allowing for timely intervention to prevent further growth. Furthermore, the use of interpositional barriers or stents is shown to reduce adhesion formation in surgical settings such as septoplasty or sinus surgery by separating opposing mucosal surfaces, which may be applied to this patient’s situation [6]. Lastly, careful intraoperative mucosal approximation and minimizing raw surface contact, combined with postoperative mobilization of the oropharyngeal structures, can decrease the risk of synechiae formation [7].

More validated data would be needed to further understand the underlying causes of synechiae formation during the healing process. As synechiae can be a probable issue in surgical procedures or trauma as postoperative outcomes, learning more about different preventative practices and treatments can lead to better outcomes for patients. Such examples include, but are not limited to, the usage of corticosteroids as it promotes collagen degradation and inhibits further fibroblast growth, which is vital in scar formation. Another solution could be the usage of botulinum toxin A by reducing muscle and skin tension, with the effect of improved wound healing with less noticeable scars [8].

## 4. Conclusion

This case report highlights the importance of understanding the healing process after a traumatic injury of the oral and nasopharyngeal mucosa to better understand postoperative complications that may come from their abnormal adhesion. Further studies could address predisposing factors that make a patient more susceptible to this formation as well as techniques and practices to help prevent complications such as in the case presented here.

## Funding

No funding was received for this manuscript. This research did not receive any grants or funding from agencies.

## Consent

Informed consent was obtained.

## Conflicts of Interest

The authors declare no conflicts of interest.

## Data Availability

Data sharing is not applicable to this article as no datasets were generated or analyzed during the current study.
